# Intelligent Ball Bearing Fault Diagnosis Using Fractional Lorenz Chaos Extension Detection

**DOI:** 10.3390/s18093069

**Published:** 2018-09-12

**Authors:** An-Hong Tian, Cheng-Biao Fu, Yu-Chung Li, Her-Terng Yau

**Affiliations:** 1 College of Information Engineering, Qujing Normal University, Qujing 655011, China; tianah@mail.qjnu.edu.cn (A.-H.T.); fucb@mail.qjnu.edu.cn (C.-B.F.); 2 Department of Mechanical Engineering, National Cheng Kung University, 1 University Road, Tainan City 701, Taiwan; a238966777@gmail.com; 3 Department of Electrical Engineering, National Chin-Yi University of Technology, Taichung 41170, Taiwan

**Keywords:** ball bearing, fractional Lorenz chaos system, Extension theory, Chua’s Circuit fault diagnosis

## Abstract

In this study we used a non-autonomous Chua’s circuit, and the fractional Lorenz chaos system. This was combined with the Extension theory detection method to analyze the voltage signals. The bearing vibration signals, measured using an acceleration sensor, were introduced into the master and slave systems through a Chua’s circuit. In a chaotic system, minor differences can cause significant changes that generate dynamic errors. The matter-element model extension can be used to determine the bearing condition. Extension theory can be used to establish classical and sectional domains using the dynamic errors of the fault conditions. The results obtained were compared with those from discrete Fourier transform analysis, wavelet analysis and an integer order chaos system. The diagnostic rate of the fractional-order master and slave chaotic system could reach 100% if the fractional-order parameter adjustment was used. This study presents a very efficient and inexpensive method for monitoring the state of ball bearings.

## 1. Introduction

The ability to accurately monitor the state of wear and performance of ball bearings [1,2,3,4,5,6,7,8] in machine tools is important for several reasons. The most serious being that an unexpected breakdown can cause irreparable damage to other parts of the machine. However, over the long term, the wear of bearings will result in machining accuracy loss, combined with poor performance reducing product quality. This makes monitoring of the state of bearings important for the early detection of problems, so that timely replacement can be made.

Many recent studies have been made into the various methods for ball bearing fault diagnosis. The methods mainly used involve stator current [9,10,11,12], audio [13,14] and vibration signals [15,16]. For signal analysis, both discrete Fourier [17] transform and wavelet [18,19,20,21,22,23,24] were the most frequent analyses used. Over the last few years, chaos systems have been extensively used for diagnosis [25,26] with the fractional-order chaos method giving better diagnostic results, than a simpler chaotic system [27,28].

**Figure 1:**
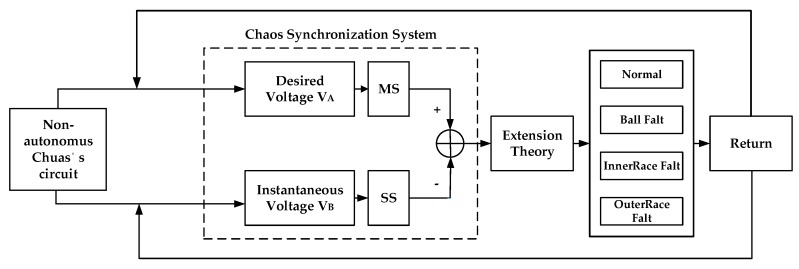
Ball bearing diagnosis system flow chart.

Although chaos system results are valid and useful, it is necessary to add signal pre-processing, as used in fractal theory, to calculate fractal dimension and lacunarity, and allow real-time diagnosis. In this paper we offer a new approach, using synchronized fractional chaos processing with Chua’s Circuit, to remove less characteristic signals and diagnose the current state of a ball bearing system. The amount of waveform data used can be downsized with a consequent reduction of calculation time. A much better diagnosis ratio can also be achieved. The bearing system signals can be analyzed by extension identification, and better order numbers can be chosen by observing the chaotic synchronizing motion traces from different fractional orders. Figure 1 shows a flow chart of the system used in this study. Details will be given in the following sections.

## 2. Chua’s Circuit

**Figure 2:**
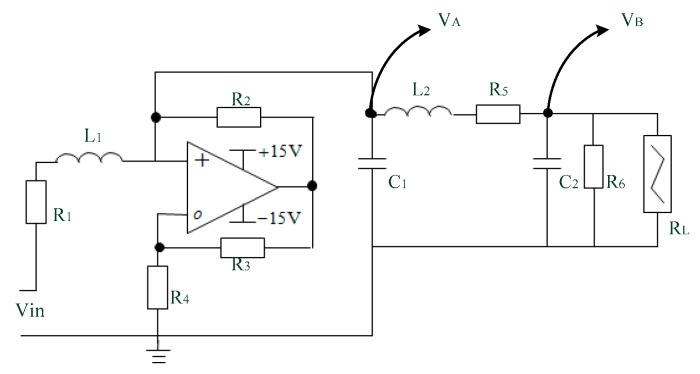
Diagram of Chua’s circuit.

The simple layout of Chua’s circuit [29,30] is shown below. The circuit has three active components: Capacitors, inductors, resistors, and a non-linear resistor. $R_{L}$ is the Chua’s diode, as shown in Figure 2. The famous Chua’s circuit is used extensively in electronics in the analysis and processing of chaotic phenomena. In this study, the Chua’s circuit is used to fill a vacancy in a fractional order chaotic system.

According to Kirchhoff’s law, the Chua’s circuit’s state equation is as Equation (1):

(1) $$\left\{ \begin{array}{l} C_{1}\frac{dV_{C1}}{dt}=-gV_{C1}+i_{L1}-i_{L2}\\ C_{2}\frac{dV_{C2}}{dt}=i_{L1}-i_{RL}\\ L_{1}\frac{di_{L1}}{dt}=-V_{C1}+i_{L1}R_{1}+V_{in}\sin(2\pi k)\\ L_{2}\frac{di_{L2}}{dt}=V_{C1}+V_{C2}+i_{L2}R_{5} \end{array}\right.$$

where *k* is a parameter corresponding to higher harmonics, and the current *i*_RL_ is defined as Equation (2).

(2) $$i_{\mathrm{RL}}=G_{a}V_{C2}+\frac{1}{2}(G_{b}-G_{a})\left[\left|V_{C2}+E_{a}\right|-\left|V_{C2}-E_{a}\right|\right]$$

wherein *G_a_* and *G_b_* are the slopes, and *E_a_* is the breakpoint.

In this study, a non-autonomous Chua’s circuit is used to record ball bearing signals, and the voltages V_a_ and V_b_ waveform characteristics. which are introduced into chaos as an extension matter-element model, using the extension algorithm.

## 3. Experiment System

**Figure 3:**
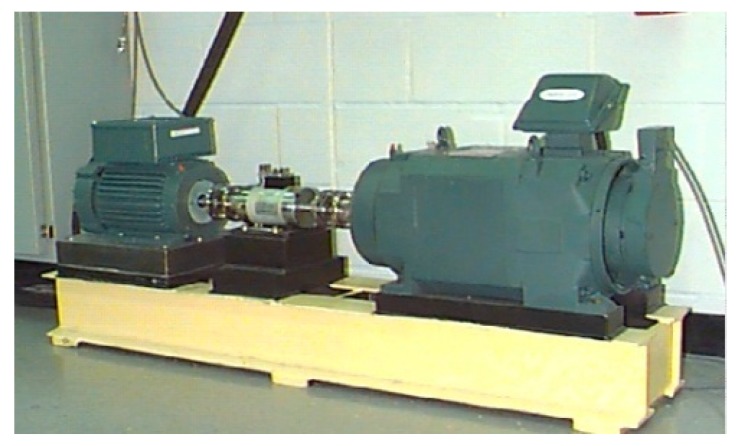
Ball bearing experiment platform.

This paper has used simulation data from the US Case Western Reserve University Bearing Data Center [31]. The database provides experimental data for both normal and faulty ball bearings. Figure 3 shows the experimental platform utilized by the database. It consists of a 2 HP (horsepower) motor, encoders, a shaft for supporting the bearings, and a dynamometer. Faults are introduced in the bearings by electro-discharge machining (EDM) on the inner ring raceway, outer ring raceway and the rolling element of 0.007, 0.0014 and 0.0021-inch diameter, to a depth of 0.011 inch for monopoint faults. Loads were applied in the range of 0–3 HP for testing. Please refer to Table 1 for detailed specifications. Accelerometers were used to collect data from faulty and normal bearings. These were installed at the ends of the actuator and motor case at the 12 o’clock position. The collected data were processed and stored using MATLAB.

## 4. Chaos Theory

Chaos theory deals with the behavior of non-linear dynamic systems which is very sensitive to small changes in initial conditions. Motion traces can be created by chaos attractors which are ordered but non-periodic. Original motion signals may be small, but will result in the output of much bigger signals. In the chaos signal synchronizing system, there is both a Master (MS) and Slave (SS) chaos system. These two systems have different initial values and this will result in two motion traces, one for each chaotic phenomenon. However, the master-slave systems can be synchronized by adding a controller to the slave system to track the master. In this section, the design of the fractional-order chaotic self-synchronization process is described in detail. Table 2 shows the definitions and notations used in the paper.

The characteristic quantities adopted in this paper are subject to the size of the natural dynamic errors between the master and slave, and master-slave synchronizing systems. This means the master-slave systems mentioned here have not been designed with controllers, and the dynamic error conditions can be acquired simply by subtracting one from the other. It is intended that the master-slave chaotic system be used to control the system and maintain its stability. The master (*MS*) and slave systems (*SS*) [32,33], can be expressed in Equations (3) and (4):

(3) $$MS=\dot{X}=AX+f\left(X\right)$$

(4) $$SS=\dot{Y}=AY+f\left(Y\right)+U$$

$X\in R^{N}$ and $Y\in R^{N}$ are state vectors, A is an N×N system matrix, the f(x) and f(y) vectors are non-linear and *U* is also a non-linear control term. Signal processing is done by a non-linear chaotic system, such as that of Lorenz [34], where N = 3 is used. The *MS* dynamic equations can be shown in matrix form:

(5) $$\begin{array}{l} \left\{ \begin{array}{l} \overset{•}{x_{1}}=\alpha \left(x_{2}-x_{1}\right)x_{1}\\ \overset{•}{x_{2}}=\beta x_{1}-x_{1}x_{3}-x_{2}\\ \overset{•}{x_{3}}=x_{1}x_{2}-\gamma x_{3} \end{array}\right.\\ \left\{ \begin{array}{l} \overset{•}{y_{1}}=\alpha \left(y_{2}-y_{1}\right)\\ \overset{•}{y_{2}}=\beta y_{1}-y_{1}y_{3}-y_{2}\\ \overset{•}{y_{3}}=y_{1}y_{2}-\gamma y_{3} \end{array}\right. \end{array}$$

The dynamic error is defined as $e_{1}=x_{1}-y_{1}$, $e_{2}=x_{2}-y_{2}$, $e_{3}=x_{3}-y_{3}$ and $e={\left[e_{1},e_{2},e_{3}\right]}^{T}$, calculation of the dynamic error function and the matrix form yields:

(6) $$\dot{e}=\left[\begin{array}{ccc} \left(1-\alpha \right) & \alpha & 0\\ \beta & 0 & 0\\ 0 & 0 & \left(1-\gamma \right) \end{array}\right]\left[\begin{array}{c} e_{1}\\ e_{2}\\ e_{3} \end{array}\right]+\left[\begin{array}{c} 0\\ -e_{3}\\ e_{2} \end{array}\right]e_{1}$$

**Figure 4:**
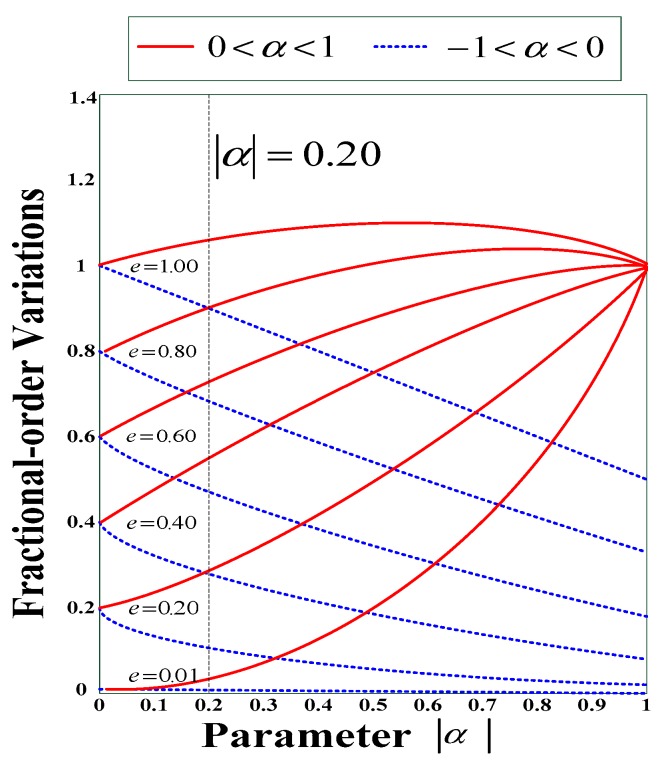
Dynamic error caused by fractional-order changes (α=±0.02,m=1).

A Grünwald-Letnikov fractional order approximation [20,24,35,36,37] gives Equation (7)

(7) $$D_{e}^{\pm \alpha }e^{m}\approx \frac{\Gamma (m+1)}{\Gamma (m+1\pm \alpha )}e^{m\pm \alpha }$$

e is the dynamic error, m a real (arbitrary) number, and α is the desired phenomenon, see Figure 4. From |α|, the following rules apply:

1. 0.00<|α|≤0.20: For arithmetic quantification as well as proportional application
2. 0.20<|α|≤1.00: For classification and control of non-arithmetical values

To express fractional changes, fractional order modifications on the first-order differential system can be expressed as in Equation (8).

(8) $$\begin{array}{c} \frac{d}{dt}\frac{d^{-\alpha }}{dt^{-\alpha }}\approx A(e)\frac{d^{-\alpha }}{dt^{-\alpha }}\left[\begin{array}{l} e_{1}^{1}\\ e_{2}^{1}\\ e_{3}^{1} \end{array}\right]+\frac{d^{-\alpha }}{dt^{-\alpha }}\left[\begin{array}{l} e_{1}^{0}\\ -e_{1}e_{3}\\ e_{1}e_{2} \end{array}\right]\\ \;\Rightarrow \left[\begin{array}{c} D^{q}e_{1}\\ D^{q}e_{2}\\ D^{q}e_{3} \end{array}\right]\approx \left[\begin{array}{ccc} -\alpha^{\prime } & \alpha^{\prime } & 0\\ \beta^{\prime } & 0 & 0\\ 0 & 0 & -\gamma^{\prime } \end{array}\right]\left[\begin{array}{c} e_{1}^{1+\alpha }\\ e_{2}^{1+\alpha }\\ e_{3}^{1+\alpha } \end{array}\right]+\left[\begin{array}{c} \frac{\Gamma (1)e_{1}^{\alpha }}{\Gamma (1+\alpha )}\\ \frac{\Gamma (1)e_{1}e_{3}e_{2}^{\alpha }}{\Gamma (1+\alpha )}\\ \frac{\Gamma (1)e_{1}e_{2}e_{3}^{\alpha }}{\Gamma (1+\alpha )} \end{array}\right] \end{array}$$

where q=(1−α), 0<q≤1 is to achieve fractional order, and Γ(•) is a gamma function. Γ(1)=Γ(2)=1, wherein the system parameters $\dot{\alpha }$, $\dot{\beta }$ and $\dot{\gamma }$ are non-zero constants. They must therefore be converted into an expression, such as in Equation (9).

(9) $$\alpha^{\prime }=\frac{\alpha \Gamma (2)}{\Gamma (2+\alpha )},\beta^{\prime }=\frac{\beta \Gamma (2)}{\Gamma (2+\alpha )},\gamma^{\prime }=\frac{\gamma \Gamma (2)}{\Gamma (2+\alpha )}$$

The chaos system used here employs a non-linear Chua’s circuit with signal transform inputs V_A_ and V_B_ and *e*_1_, *e*_2_, and *e*_3_, to make trace diagrams of the phase domain. The important characteristics are the four bearing state signals: Normal signal, bearing fault, inner ring fault and outer ring fault. The dynamic errors are:

(10) $$\left\{ \begin{array}{l} e_{1}\left[i\right]=x\left[i\right]-y\left[i\right]\\ e_{2}\left[i\right]=x\left[i+1\right]-y\left[i+1\right]\\ e_{3}\left[i\right]=x\left[i+2\right]-y\left[i+2\right] \end{array}\right.$$

where x and y are the V_A_ and V_B_ signals.

The chaos system employed in this study was used for the diagnosis of ball bearing faults by the analysis of vibration signals. The data were downloaded from the Center website, as previously mentioned. Originally around 240,000 entries were collected at a frequency of 48k. This included about 48,000 from the motor startup transient state, which were discarded. The remainder was divided into two, each including about 96,000 entries. One was used for analysis, and the other for the result verification. The trajectories of the dynamic errors $e_{1}{,e}_{2}{,e}_{3}$ formed key characteristics, according to the phase planes and signals representing four different bearing conditions: Normal, ball fault, fault in the inner race and in the outer race. The signals resulting from dynamic errors were acquired by the introduction of V_A_ and V_B_ test signals into the master synchronization (MS) system. The *e*_2_ and *e*_3_ dynamic error signals were used for plotting the trace diagrams. The trajectories existing for each state were used to establish a matter-element model. The signals emanating from the output of the monitoring system were identified using an extension theory.

**Figure 5:**
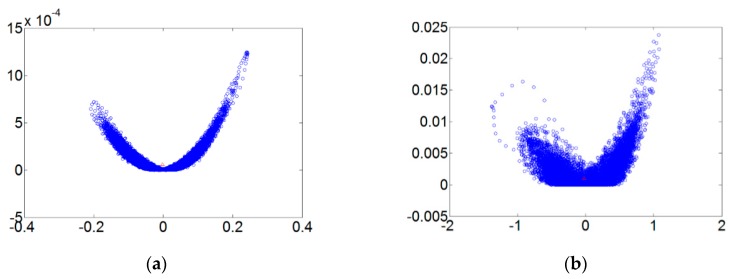

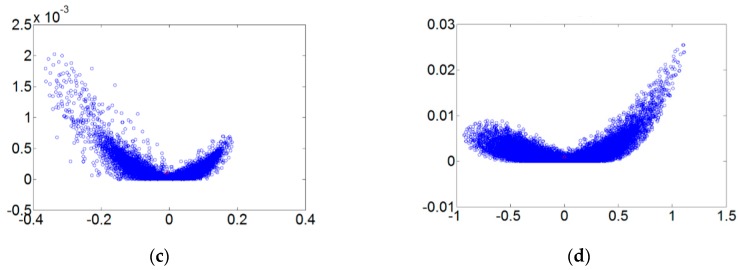
The order 1 fault dynamic error trace diagrams for all fault diameters. (**a**) Normal state; (**b**) fault diameter = 0.007; (**c**) fault diameter = 0.014; (**d**) fault diameter = 0.021.

**Figure 6:**
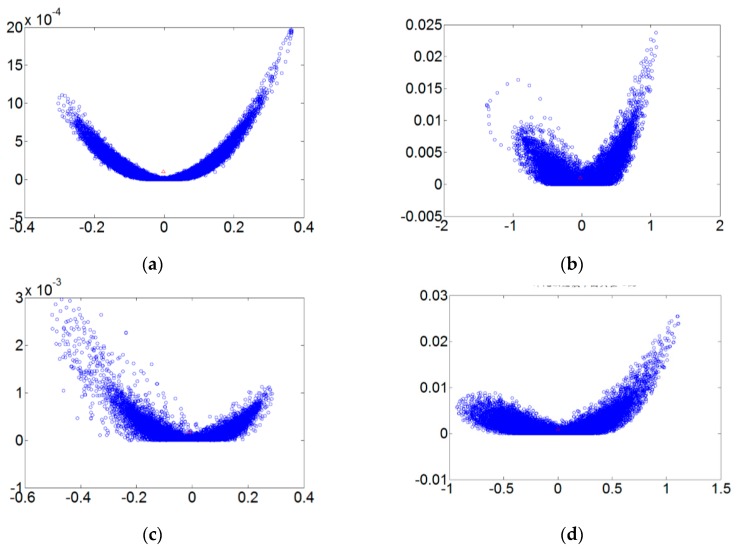
The order 0.9 fault dynamic error trace diagrams for all fault diameters. (**a**) Normal state; (**b**) fault diameter = 0.007; (**c**) fault diameter = 0.014; (**d**) fault diameter = 0.021.

**Figure 7:**
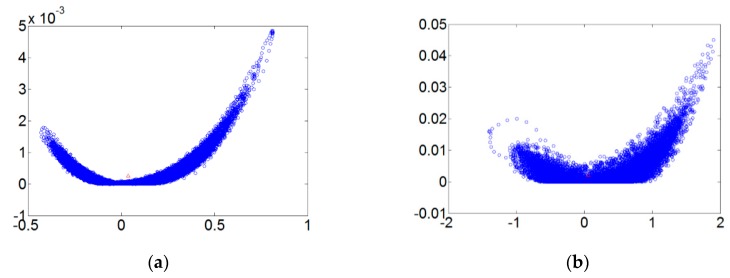

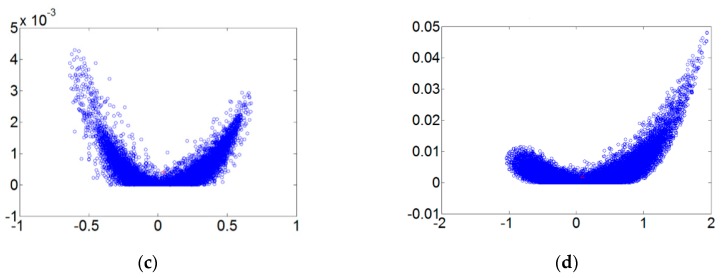
The order 0.7 fault dynamic error trace diagrams for all fault diameters. (**a**) Normal state; (**b**) fault diameter = 0.007; (**c**) fault diameter = 0.014; (**d**) fault diameter = 0.021.

**Figure 8:**
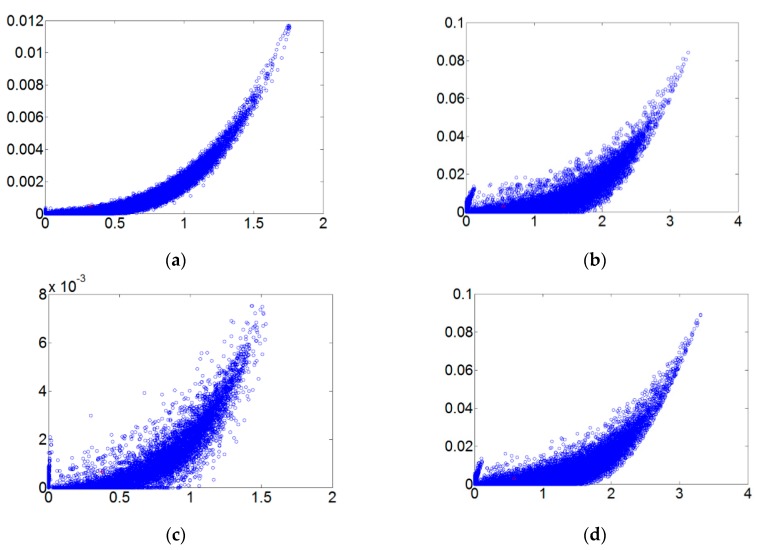
The order 0.5 fault dynamic error trace diagrams for all fault diameters. (**a**) Normal state; (**b**) fault diameter = 0.007; (**c**) fault diameter = 0.014; (**d**) fault diameter = 0.021.

**Figure 9:**
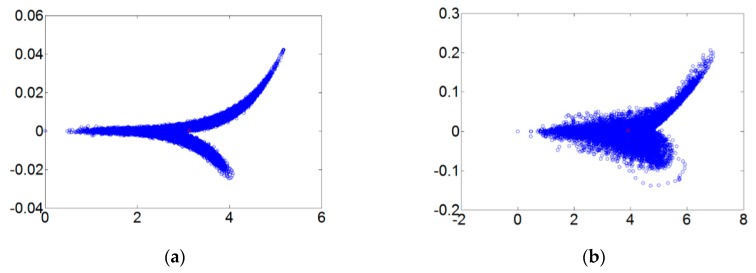

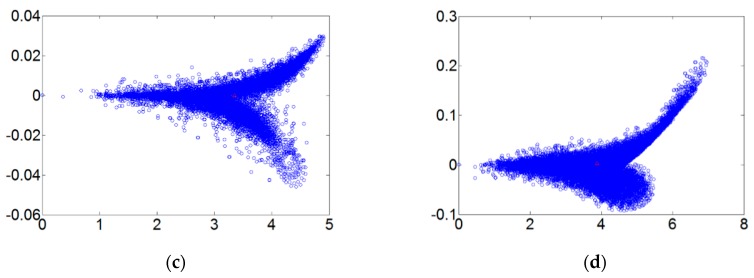
The order 0.3 fault dynamic error trace diagrams for all fault diameters. (**a**) Normal state; (**b**) fault diameter = 0.007; (**c**) fault diameter = 0.014; (**d**) fault diameter = 0.021.

Figure 5 refers to the fault diameters 0.007, 0.014, 0.021 inches and normal signals. They have been substituted using the integer order chaos system to manage the ball bearing dynamic error diagrams. Under the integer order chaos system, the dynamic errors of different states would be too concentrated in the same regions, and the characteristics would not be obvious. This means misjudgment could easily occur during the dynamic error distribution calculation of the extension theory matter-element models. Such order numbers are unsuitable for signal processing by this system. Figure 6, Figure 7, Figure 8 and Figure 9 refer to the dynamic error trace diagrams in which the fractional-order chaos system is used for processing the ball bearing signal. According to the simulation results for the fault diameters of 0.007, 0.014 and 0.021 inches, compared to the order 0.9, 0.7, 0.5 and 0.3 of the integer order chaos system, the dynamic errors among the states are more decentralized, and the trace diagram for order 0.3 is more characteristically different. This is such that, during the calculation of the dynamic error distribution, excellent identification results are provided; order 0.3 is more suitable therefore, for processing signal in the flow chart of Figure 1.

## 5. Extension Theory

**Figure 10:**
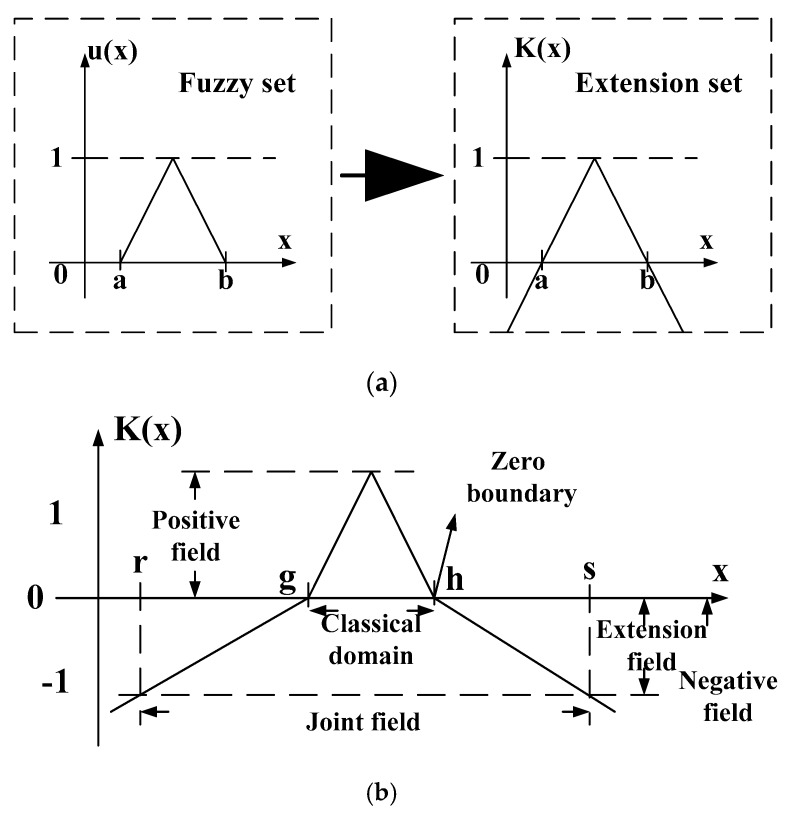
Fuzzy and extension sets. (**a**) Relationship of a fuzzy set to an extension set; (**b**) extension set diagram.

The extension theory, fully described by Su and Zhao [38], was derived from the observation of diverse conditions in various objects. and seeking regularity using their extensibility. Finally, mathematical operations were used to characterize conditions. The extension theory can generally be divided into two parts: The matter-element model and extension set which can both be used to quantify objects according to their similarity. The model allows for easy description of the images and objects. The fuzzy set [39,40] range can be extended from (0, 1) to (−∞,∞). See Figure 10 for an illustration of the fuzzy and extension sets. Wang et al. [41,42] give a more detailed description of the matter-element theory and the extension set.

### 5.1. Matter-Element Theory

A study of properties is the essence of the matter-element theory and among these are change, transformation and extensibility. Various objects can be distinguished by their specific characteristics and used in an analysis that clearly indicates any differences between them. Differences in object position, form, or mode can be conveyed by values in mathematical terms, more specifically by a matrix (11):

(11) $$\emptyset_{i}={\left[\begin{array}{ccc} O_{i} & \varepsilon_{i} & \mu_{i} \end{array}\right]|}_{i=1,2,\ldots ,N}$$

*N* is the matter-element, *ε* the matter-element characteristics and *μ* the characteristics value. In an object with n characteristics, the vector of the characteristics can be expressed by its corresponding value, referred to as an n-dimension matter-element.

When these values are spread across a particular range, this is referred to as a classical domain and is part of a joint domain. Assuming intervals of $F_{0}=(g,h)$ and F=(r,s), where: $F_{0}\in F$, *g* and *h* are the high and low limits of a classical domain, and *r* and *s* are the high and low limits of the joint domain.

### 5.2. Extension Sets

Sets and correlation functions are the essence of mathematical extension and serve to extend a specific set into a continuous value range (−∞,∞) expressing object properties by means of a correlation function. The set is within a range of real numbers (−∞,∞) and conveys the degree to which the features of an object are of interest. The concept of an extension set and its definitions are:

Take *Ω* to be the universe of discourse, and any element such as ω in *Ω* (i.e., *ω ∈ Ω*) has a related real number (i.e., K(ω)∈(−∞,∞)). An extension set can be defined as in Equation (12):

(12) $$\prod =\prod^{+}\cup \prod^{0}\cup \prod^{-}$$

$\prod^{+}$, $\prod^{0}$ and $\prod^{-}$ are positive, zero and negative domain of the extension set. See Figure 10 and Equations (13)–(15):

(13) $$\prod^{+}=\{\left(\omega ,y\right)|\;\omega \in \Omega ,\;y=K(\omega )>0\}$$

(14) $$\prod^{0}=\{\left(\omega ,y\right)|\;\omega \in \Omega ,\;y=K(\omega )=0\}$$

(15) $$\prod^{-}=\{\left(\omega ,y\right)|\;\omega \in \Omega ,\;y=K(\omega )<0\}$$

An examination of Figure 10 shows that, as an object gets closer to the classical domain, its correlation function becomes greater in value, and the more likely it is that the data will fall into that class. On the other hand, the value of the correlation function will become smaller the further away the object moves from the classical domain, and the less likely it is to fall into that class.

### 5.3. An Extension Theory Matter-Element Model

The establishment of a model for a mathematical problem causes it to become idealized. The question now arises as to whether the model might differ from the problem. Extension theory uses a matter-element model to deal with this issue. In extension, *O* refers to an object, with characteristics ε, and corresponding values μ. These elements are the essential matter-element factors and describe the object. Equation (16) can be used to express an object with multiple characteristics:

(16) $$\emptyset =\left[\begin{array}{ccc} O & \varepsilon_{1} & \mu_{1}\\ & \varepsilon_{2} & \mu_{2}\\ & \vdots & \vdots \\ & \varepsilon_{N} & \mu_{N} \end{array}\right]$$

Classification using condition equations is intuitive: Should a fault test point lie within the range of a certain condition, it belongs to that specific state. If a test point lies outside any condition, it will not be possible to use a condition-based method to correctly identify the fault state, and a wrong diagnosis is the most likely outcome. The extension theory and condition-based classification method differs from others, such as the fuzzy theorem, in that it emphasizes the amount of correlation using distance. A system that is based on extension theory, will automatically determine the nature of the fault at a specific point by finding the fault state to which the point in question lies closest.

In this study we used the extension theory for identification in backend detection of the FOCLCS system. Signals indicative of the present state of a bearing were classified using the amount of correlation. A high correlation value meant the matter-element object under test was close to the classical field and would fall within the related class.

## 6. Experiment Results

The fractional Lorenz chaos synchronous dynamic error system was utilized to make the ball bearing signals transform. Dynamic trace diagrams of *e*_1_, *e*_2_ and *e*_3_ have been made from observations. The different states provided by the experimental database include normal, outer ring, roll ring and inner ring faults. Extension matter-element models were designed for distinguishing intelligent monitoring output systems which work on a 48 k (Hz) base sampling rate. One second data volumes are formed from observation of important characteristics of the different state dynamic errors, *e_2_* and *e_3_*.

In the establishment of the extension matter-element model, four characteristic items, $\alpha_{1}$, $\alpha_{2}$, x and y were used as a basis for judging the state of a ball bearing. $\alpha_{1}$ represents the left-half distribution area of the horizontal axis of the dynamic error trace diagram, $\alpha_{2}$ the right-half distribution area of the horizontal axis of the dynamic error trace diagram. x stands for the left-half distribution area of the dynamic error trace diagram, and y stands for the right-half distribution area of the dynamic error trace diagram. Equation (17) is the fault matter-element model of a ball bearing at 0 HP for all diameters.

(17) $$\begin{array}{l} \left[\begin{array}{ccc} Normal & \alpha_{1} & \left[0,2\right]\\ & \alpha_{2} & \left[2,4\right]\\ & x & \left[-0.01,0.05\right]\\ & y & \left[-0.01,0.03\right] \end{array}\right]\left[\begin{array}{ccc} 0.007 & \alpha_{1} & \left[0,2\right]\\ & \alpha_{2} & \left[2,4\right]\\ & x & \left[-0.05,0.045\right]\\ & y & \left[-0.09,0.15\right] \end{array}\right]\\ \left[\begin{array}{ccc} 0.014 & \alpha_{1} & \left[0,2\right]\\ & \alpha_{2} & \left[2,4\right]\\ & x & \left[-0.02,0.01\right]\\ & y & \left[-0.021,0.02\right] \end{array}\right]\left[\begin{array}{ccc} 0.021 & \alpha_{1} & \left[0,2\right]\\ & \alpha_{2} & \left[2,4\right]\\ & x & \left[-0.05,0.03\right]\\ & y & \left[-0.05,0.15\right] \end{array}\right] \end{array}$$

**Figure 11:**
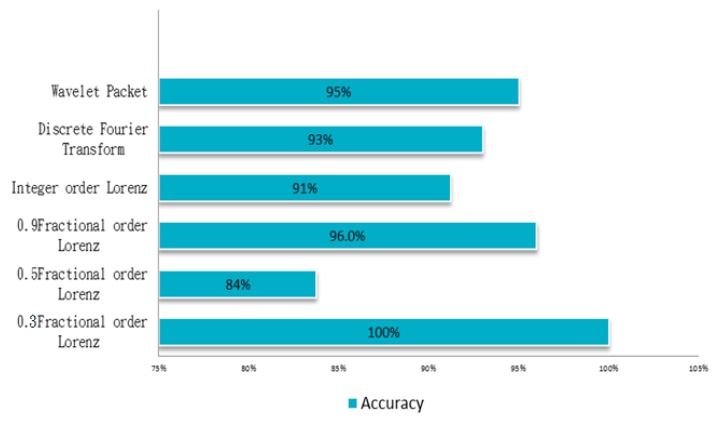
The diagnostic accuracy obtained with each different method.

The matter-element model established in this paper has four characteristics, each having a 0.25 weight setting. Each characteristic goes through extension calculations and is accurately identified for ball bearing condition. In this paper discrete Fourier transform, wavelet packet analysis, different order ranking of fractional chaos systems, and other methods were used to identify random data. The statistical method used, collected data on each state from 20 ball bearings and was used to calculate the likelihood of accurate judgment. An examination of Figure 6, Figure 7, Figure 8 and Figure 9 clearly shows that the order 0.3 fractional Lorenz chaos system was superior to other methods and orders. In addition, another advantage is that only vibration signals need to be collected for processing, and the cost of the sensors is minimal. Figure 11 shows the accuracy of results from each method used with a model built from the data of 20 tests. A total of 500 random readings (125 for each state) were used to test our diagnosis system. The results showed that when the fractional order parameter was 0.3, the accuracy rate approached 100%. However, with a fractional order of 0.5, accuracy was only 84%.

## 7. Conclusions

In this paper a method was presented for the evaluation of faults in ball bearings integrating fractional-order chaos synchronization dynamic error and extension theory. Implementation of such a ball bearing intelligent state monitoring system can be done using the LabVIEW human–machine interface. The results of our experiments show the method has some distinct advantages in that cost is low because only one sensor is needed, calculations can be made quickly, and the accuracy of diagnosis is good. This method can be used to monitor the ball bearings in every part of a machine tool. Such an intelligent system would detect problems fast and accurately, making it possible for faulty bearings to be found and replaced before a breakdown. This will improve the overall efficiency of any machine tool installation.

In the system used in this study, a Chua’s circuit was added to a fractional order chaotic system to pre-process the vibration signals. Extension was then used to identify the fault state. If the pre-processing was not good enough, the extension theory did not give good results. The adjustment could be made to the fractional-order parameter. Although the accuracy achieved was close to 100%, we still need to be sure that 0.3 is the best order for all ball bearing systems in future. Furthermore, the database used in this study only considered changes in axial load at a set speed. Experiments with different axial load over a range of speed need to be done in the future.

This system can record and store captured signals and diagnosis results. If it could be integrated with Ethernet control automation technology (EtherCAT), this might helpful in several ways. For example, the data could be uploaded to the cloud and immediately be available for other users anywhere. Such a database would be extremely useful as a check for review and evaluation, or the improvement of existing methods. It would certainly help the development of Industry 4.0.

## Figures and Tables

**Table 1:** Types of ball bearing faults.

Sampling Frequency (Hz)	Motor Load (HP)	Fault Single Point Diameter (inches)	Fault Single Point Depth (inches)	Fault Condition
12 k 48 k	0 1 2 3	0.007 0.014 0.021	0.011	Normal ball bearing fault inner ring fault outer ring fault

**Table 2:** Nomenclature.

Notation	Definition	Notation	Definition
*X*	The system states of the master system	*Г( )*	Gamma function
*Y*	The system states of the slave system	*a’, b’, c’*	System parameters of fractional–order system
*f*	Non-linear function	*Ф_i_*	Dynamic error equation
*U*	Control input	*g, h*	The upper and lower limits of the classical domain
*A*	System parameter vector	*r, s*	The upper and lower limits of the joint domain
*a, b, c*	System parameters of the Chen-Lee Chaos System	*О*	The name of a matter-element
*e*	System error state vector	*ε*	The characteristics of the matter-element
*D*	Differential operator	*μ*	The values corresponding to the characteristics
*α*	The value of differential order	*Ω*	The universe of discourse
